# Effect of Behavior Change Communication on Self-Care Practices Among Adult Type-2 Diabetic Patients in a Semi-urban Community of South India: A Quasi-Experimental Study

**DOI:** 10.7759/cureus.38805

**Published:** 2023-05-09

**Authors:** Sreenivasulu M Ganji, Praveen Kumar BA, Devi Varaprasad M, Janakiraman Pichandi

**Affiliations:** 1 Department of Pathology, Osmania Medical College, Hyderabad, IND; 2 Department of Community Medicine, PES Institute of Medical Sciences and Research, Kuppam, IND; 3 Department of Community Medicine, SRM (Sri Ramaswami Memorial) Medical College Hospital and Research Centre, Chennai, IND

**Keywords:** glycemic control, health education, community-based, self-care practices, behaviour change communication, diabetes mellitus

## Abstract

Background: Diabetes mellitus is often termed the modern epidemic, and India ranks second after China in the global disease burden. Practice and adherence to essential self-care behaviors, positively correlated with good glycemic control and reduced complications in people with diabetes, have been inadequately understood, especially in a semi-urban setting.

Methods: This community-based interventional study was done among 269 known adult type 2 diabetic patients from a semi-urban community in South India for three months. By simple random sampling, known diabetics identified in the health survey by the tertiary care teaching institute were considered for the study. Self-care practices in diabetes were recorded in the pre-test using a validated semi-structured questionnaire. Two health education sessions, each for 30 minutes, were conducted with 15-20 subjects in a group. Health education materials on self-care in diabetes, such as charts, handouts, video clippings, and PowerPoint presentations in the local language, were used. The self-care practices were re-recorded in the post-test after two months. Inferential statistics were carried out with a t-test, analysis of variance (ANOVA), and Pearson correlation coefficient, and a p-value less than 0.05 was considered statistically significant.

Results: A total of 253 diabetic subjects were included in the final analysis, with an attrition rate of 6%. The mean age of participants was 56.5 ± 11.9 years. The mean score of self-care practices among diabetic subjects at the baseline was 14.6 ± 13.2. Illiteracy and smoking habit were significantly associated with lower self-care scores in the pre-test. In the post-test, after health education, there was a significant improvement in the mean self-care practices score and a reduction in the mean fasting blood sugar level. Also, a significant mild negative correlation between the self-care scores and blood sugar levels was seen (Pearson correlation coefficient = -0.21, p < 0.001).

Conclusion: Self-care practices, which were not satisfactory in most diabetic participants, were found to be significantly impacted by the small group education. This stresses the need for effective health education sessions as envisaged under the national program.

## Introduction

Diabetes mellitus is a chronic disease with impaired insulin production or utilization in the body. It is often termed the modern epidemic and has a prevalence of 10.4% in India. As per our population, one in six people with diabetes is Indian. India has 77 million diabetics, second to China. India has more than one million deaths from diabetes and its complications [1,2]. This increase in diabetes prevalence in the last two decades has resulted from rapid cultural and social changes, including aging populations, increasing urbanization, dietary changes, reduced physical activity, and other unhealthy behavior [3].

With the decline in infectious diseases, lifestyle diseases gain more importance due to their ability to cause more significant complications and economic drain on the population. Treatment of complications costs 50% of the direct cost of diabetes. An extra 35% is added to the global diabetes expenditure due to indirect costs like premature deaths, disabilities, drop-outs, and absenteeism [4].

Successful management of diabetes requires the adoption of numerous behaviors and skills [5]. Self-care is the activities people perform independently to maintain life, health, and well-being [6]. Seven essential self-care behaviors in people with diabetes have been positively correlated with good glycemic control and reduced complications. These seven essential self-care behaviors are healthy eating, being physically active, monitoring blood sugar, complying with medications, good problem-solving skills, healthy coping skills, and risk-reduction behaviors [7].

Health belief model (HBM) is a well-recognized health behavior change communication model. Susceptibility of the target population, identification of the seriousness of the problem, and belief in the benefit of prevention are the essential parts of HBM that focus on health motivation. The social cognitive theory (SCT) is based on the continuous interaction between personal, environmental, and behavioral factors [8]. These models can be used to design educational interventions for diabetes.

With about 87% of all deaths due to diabetes occurring in low- and middle-income countries, preventing complications is a significant pillar in diabetes treatment [3]. Despite this, compliance or adherence to these activities is low, especially when considering long-term changes [7]. Awareness and education play an essential role in compliance with self-care activities.

Practice and adherence to essential self-care behaviors have been inadequately understood, especially in a semi-urban setting. In this context, this study was planned to determine the prevalence of self-care practices and evaluate the impact of health education on diabetic patients' self-care practices.

## Materials and methods

**Figure 1:**
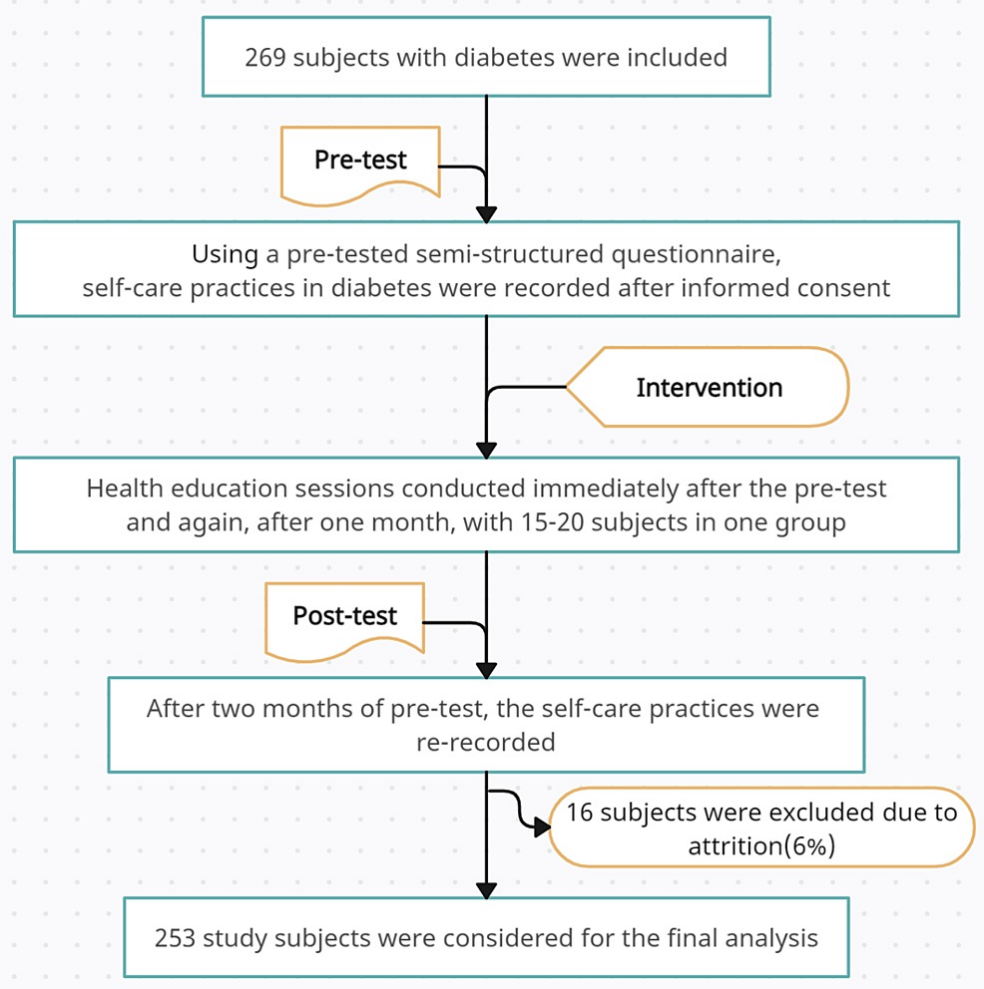
Design outline and data flow

A community-based quasi-experimental study was conducted in a semi-urban community in South India, a field practice area of a tertiary care teaching institute. The study population included adult diabetic patients and was conducted for three months. The outline of the design and data flow is depicted in Figure 1.

Inclusion and exclusion criteria

All known adult, type 2 diabetic patients of either gender who were on treatment were included. People with diabetes with acute exacerbation and a persistent hyperglycemic state, foot ulcers, hearing and visual impairment, severe comorbid illness, and those on steroid intake were excluded.

Sample size and sampling technique

Based on a prevalence of 58.4% in the self-care domain [7] with an error of 10%, a sample size of 269 was required. All the diabetics identified in the Kuppam Health Survey 2017 [9] conducted by the tertiary care teaching institute were considered for the study. Among these people with diabetes, those residing in the semi-urban area, which belongs to a field practice area of the tertiary care teaching institute, were selected by simple random sampling. Those fulfilling the inclusion and exclusion criteria were included in the study.

Intervention

The intervention was based on the theories of HBM and SCT. Appropriate health education materials on self-care in diabetes, such as charts, handouts, video clippings, and PowerPoint presentations in the local language, were prepared based on the responses from the pre-test. Health education sessions for 15-20 subjects as a group for about 30 minutes were conducted by a doctor and medico-social worker team at the urban health center. This was followed by a question-and-answer session for 10-15 minutes to clarify any doubts. Finally, 25-30 minutes were allotted to ask the participants to volunteer their experience with diabetes or to discuss their plans to adopt a self-care behavior briefly. These health education sessions were conducted for around 15 days till the total study subjects were covered. The subjects were contacted two times again, i.e., one month and two months after the pre-test, and the data regarding self-care was re-recorded in the post-tests. Glucometer-based blood sugar levels of the subjects were recorded during the visits by a doctor. During this time, the investigating doctor encouraged participants to follow self-care practices. Another health education session was conducted after the post-test to reinforce the prevention ideas.

Self-care practices

Diet practices include consuming low glycemic foods, a fiber-rich diet, regular intake of green leafy vegetables, smaller portions and multiple meals, and decreased intake of saturated fatty acids. Moderate-intensity physical activity of brisk walking, swimming, or cycling for at least 30 minutes five times a week was considered. Foot care includes avoiding barefoot walking, regularly checking footwear for injury, and checking the healing condition of wounds. Glycemic control can be achieved through regular blood glucose monitoring and follow-ups. Practices also include taking appropriate, adequate, and regular intake of medications as per their prescription. Smoking and alcohol control habits were also recorded.

Study materials

Proforma containing a pre-tested, semi-structured questionnaire having two parts was used. Part A consists of questions regarding personal identification and sociodemographic details. Part B included a questionnaire to assess self-care practices in diabetes based on the "summary of diabetes self-care activities scale" [10] with one additional item on medications. The maximum possible score is 71. Health education materials include charts, handouts, video clips, and PowerPoint presentations.

Data analysis

The data were entered into MS Excel 2016 and analyzed using Epi info software version 7. For descriptive analysis, the categorical variables were summarized using percentages, while the continuous variables were calculated by the mean and standard deviation. For inferential analysis, t-tests and analysis of variance (ANOVA) were used to determine the mean and standard deviation of sociodemographic and biochemical variables at baseline and the self-care scores. Paired t-test was used to find out the difference in self-care practices among study participants before and after intervention and also compares the means of fasting blood sugar, body mass index, and self-care scores in the pre- and post-intervention tests. The Pearson correlation coefficient was used to find the correlation between increased self-care scores and reduced blood sugar levels. A probability value < 0.05 was considered statistically significant.

Ethical statement

Ethical clearance was obtained from the Institutional Human Ethics Committee (PESIMSR/IHEC/40/2018). Informed consent was obtained from participants before enrolment into the study.

## Results

Among 253 diabetic subjects in the final analysis, males and females (134 and 119, respectively) were almost equally distributed. The mean age of participants was 56.5 ± 11.9 years. The mean score of self-care practices among diabetic subjects at the baseline was 14.6 ± 13.2. Table 1 summarizes the sociodemographic and biochemical variables at baseline and the self-care scores. It can be noted from Table 1 that illiterate participants and smokers had significantly lower self-care scores. There was no significant difference in the mean self-care scores among the other categories.

**Table 1:** Demographic and biochemical variables with the self-care score in the pre-test (n = 253) ^t-test #ANOVA (analysis of variance) *p < 0.05 is significant SD: Standard deviation; BMI: Body mass index.

Demographic and biochemical variables	Number (%)	Self-care score (Mean ± SD)	p-value (t-test/ANOVA)
Age in years			
30-60	133 (52.6%)	13.24 ± 11.43	0.088^
60 & above	120 (47.4%)	16.12 ± 14.89	
Gender			
Male	134 (52.9%)	14.97 ± 12.17	0.636^
Female	119 (47.1%)	14.19 ± 12.14	
Literacy			
Illiterate	149 (58.9%)	9.6 ± 9.9	<0.001^#*^ Post-hoc: significant in all groups
Primary	84 (33.2%)	19.0 ± 12.6	
Secondary	20 (07.9%)	33.8 ± 13.6	
BMI			
Underweight	21 (08.3%)	12.9 ± 12.4	0.368^#^
Normal	128 (50.6%)	15.26 ± 12.7	
Overweight	75 (29.6%)	11.53 ± 13.3	
Obese	29 (11.5%)	15.6 ± 13.4	
Fasting blood sugar			
≤130 mg/dL	75 (29.6%)	16.5 ± 15.8	0.224^
>130 mg/dL	178 (70.4%)	13.9 ± 12.1	
Smoking			
Present	26 (10.3%)	10.23 ± 10.2	0.033^^*^
Absent	227 (89.7%)	15.11 ± 13.4	

Prevalence of self-care practices

A good general diet (balanced diet) was practiced by 9.48% of participants on most days of the week. In comparison, good specific diet practices were seen in 3.55% of those who consumed fruits and vegetables and 9.88% of those who avoided saturated fats on most days of the week. Only 9.48% of participants practiced specific exercises, and 11.85% reported general physical activity for the prescribed duration on most days. Blood sugar testing was reported by only 3.16% of participants on most days in the preceding week, while 47% reported having done foot inspections and 51.78% reported wearing footwear six to seven days a week. About 56.13% of the participants reported compliance with medication in six to seven days.

Effect of intervention

Changes in the various self-care practices after intervention are listed in Table 2, which compares the response days reported in the pre-test and the post-test questionnaires. Three people reported attempting to stop smoking/tobacco use in the past month. Overall, except for the blood sugar levels, which required going to a healthcare facility, most practices showed significant improvement after health education and participant discussions. This can be seen in the increased mean self-care score of 46.74 ± 11.11.

**Table 2:** Self-care practices among study participants before and after intervention (n = 253) Percentages are within parentheses. *p < 0.05 = statistically significant.

Self-care practice	0-1 day/week, n (%)		2-5 days/week, n (%)		6-7 days/week, n (%)		Mean score (days/week)		p-value
	Pre-test	Post-test	Pre-test	Post-test	Pre-test	Post-test	Pre-test	Post-test	
Healthful eating plan in last week	199 (78.66)	024 (9.48)	030 (11.85)	006 (2.37)	024 (9.48)	223 (88.14)	1.86 ± 2.04	5.91 ± 2.09	<0.001*
Healthful eating plan in last month	197 (77.86)	023 (9.09)	032 (12.64)	013 (5.14)	024 (9.48)	217 (85.77)	1.06 ± 2.13	5.81 ± 2.10	<0.001*
Fruit and vegetable consumption	208 (82.21)	058 (22.92)	036 (14.23)	072 (28.46)	009 (3.55)	123 (48.62)	0.71 ± 1.44	4.69 ± 2.75	<0.001*
High-fat foods consumption	025 (9.88)	016 (6.32)	014 (5.53)	067 (26.48)	214 (84.58)	170 (67.19)	1.23 ± 2.22	5.69 ± 2.02	<0.001*
General physical activity	205 (81.03)	054 (21.34)	018 (7.11)	026 (10.27)	030 (11.85)	173 (68.38)	1.05 ± 2.27	5.32 ± 2.83	<0.001*
Specific exercises	212 (83.79)	055 (21.74)	017 (6.72)	031 (12.25)	024 (9.48)	167 (66.01)	0.95 ± 2.16	5.23 ± 2.85	<0.001*
Blood sugar testing	225 (88.93)	232 (91.7)	020 (7.91)	018 (7.11)	008 (3.16)	003 (1.19)	0.69 ± 1.44	0.79 ± 1.04	0.33
Foot inspection	129 (50.98)	015 (5.93)	005 (1.97)	0 (0)	119 (47.03)	238 (94.07)	3.32 ± 3.46	6.59 ± 1.66	<0.001*
Using chappals	117 (46.24)	15 (5.93)	5 (1.97)	0 (0)	131 (51.78)	238 (94.07)	3.68 ± 3.46	6.59 ± 1.66	<0.001*
Medication adherence	099 (39.13)	012 (4.74)	012 (4.74)	005 (1.97)	142 (56.13)	236 (93.28)	4.07 ± 3.37	6.66 ± 1.46	<0.001*

**Figure 2:**
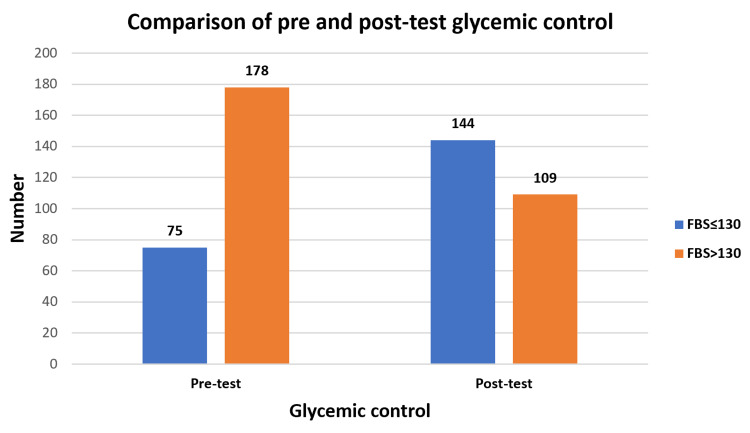
Comparison of glycemic control before and after educational intervention (n = 253) FBS: Fasting blood sugar (mg/dL).

**Figure 3:**
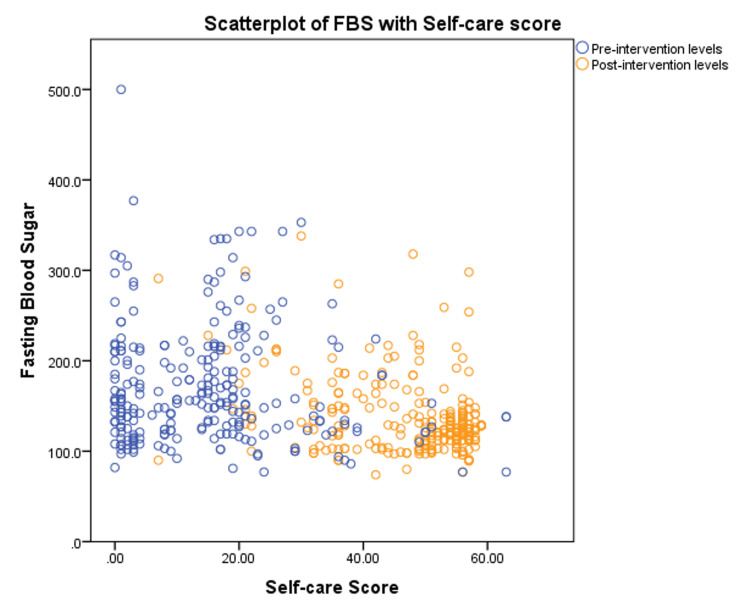
Comparative scatterplot of pre- and post-intervention blood sugar levels across self-care scores FBS: Fasting blood sugar (mg/dL).

Table 3 compares the means of fasting blood sugar, body mass index, and self-care scores in the pre- and post-intervention tests. In the post-test, after health education, there was a significant reduction in the mean fasting blood sugar level, and the mean self-care practices score improved. Accordingly, it was also observed that the number of people with good glycemic control, i.e., fasting sugar levels of ≤130 mg/dL, increased from 75 study subjects in the pre-intervention baseline to 144 subjects in the post-intervention study (Figure 2). In the post-intervention study, there was a significant mild correlation between the increase in self-care scores and a reduction in blood sugar levels (Pearson correlation coefficient = -0.21, p < 0.001). The scatterplot of fasting blood sugar (FBS) against self-care scores for both pre- and post-intervention studies is depicted in Figure 3.

**Table 3:** Impact of health education on self-care practices, fasting blood sugar, and body mass index (n = 253) *p < 0.05 = statistically significant. SD: Standard deviation.

Factors	Mean ± SD	p-value
Fasting blood sugar (mg/dL)		
Pre-test	171.05 ± 64.65	<0.001*
Post-test	137.91 ± 43.14	
Body mass index (kg/m^2^)		
Pre-test	24.12 ± 4.53	0.412
Post-test	24.37 ± 5.17	
Self-care practice scores		
Pre-test	14.61 ± 13.23	<0.001*
Post-test	46.74 ± 11.11	

## Discussion

Diabetes is a growing public health concern, and its prevalence is steadily increasing in India [1,11]. We designed this educational intervention based on a HBM and social cognitive behavior change theory. Unlike individual education, a group-based education of 15-20 members provides direct opportunities for people to learn from peers, be supported by peers, compare themselves with others in the same situation, socialize, and feel more involved while being helpful to others. This study found that the self-care practices followed by the subjects were variable and depended on the specific category, with just 47%-56% of people having good compliance for medication intake and foot care practices at the pre-intervention level.

This study had a direct relationship between literacy and self-care score in both stages. This would indicate that literate diabetics had better self-care practices and would adopt better health practices sooner. The self-care score was not related to BMI. Also, as the study lasted only two months, there was no significant change in BMI or smoking habits. There was a more direct relationship between blood sugar levels and self-care scores than other demographic factors because of health education on diet and physical activity. Smoking was also found to be significantly associated with lower self-care scores.

We noticed that 78%-82% of participants had healthy eating habits less than two days a week, while 84% had a high-fat diet more than five days a week. Physical activity was similarly low in 81%-83% of the participants, while blood sugar testing was performed less than two days a week by 89% of them. Only 47%-51% had good foot care practices more than five days a week, while only 56% had medication adherence more than five days a week. These numbers were comparable to various studies in India by Gopichandran et al., Dasappa et al., Karthik et al., Ravi et al., and Rajasekharan et al. [12-16]. A Malaysian study by Kueh et al. reported better diet and foot care scores.

In contrast, the exercise and glucose testing scores were poor [17]. An Iranian study by Modarresi et al. reported that foot care was poor among those with a better self-care score; however, this association was not significant [18]. A Grecian study by Akritidou et al. reported similar foot care and exercise numbers [19]. It shows that self-care is an often-neglected part of diabetes management, probably due to a lack of self-care health education. There needs to be a proactive effort from healthcare professionals to address this gap to ensure better health quality for diabetes care.

Post-intervention, it was noted that there was a significant increase in compliance days, except for blood sugar testing across all practice subgroups. As most testing is done at a laboratory and is advised once in one to three months, this might explain the finding. Although 67% still consumed a high-fat diet most days of the week, it was less than the pre-intervention level of 84.58%. Fruit and vegetable consumption increased from 4% to 49%, and physical activity increased from 10%-12% to 66%-68% on most days. While this is good, 21%-22% of participants consume fruits and vegetables and do physical exercises less than two days a week. Overall, this is similar to the improvement seen by Akritidou et al. in their study [19]. A study from China by Zheng et al. also explored the benefits of two-session education programs for self-management of diabetes. Although the sessions were conducted two days apart, the study compared this with a control group after three months and included practical sessions [20]. Iranian research by Ahrari et al. reported that, following a 10-session self-care training workshop, a comparison of parameters after six months of intervention showed that the mean serum HbA1C level decreased significantly, and the mean scores of the quality of life increased significantly in the experimental group [21]. In another Iranian study by Esferjani et al., three online educational sessions by mobile phones were given to participants in the interventional group, and results showed that there was a significant increase in the mean self-care scores, with a significant reduction in the mean serum HbA1C levels in the interventional group [22]. Further studies on educational interventions are required for diabetes care in Indian subpopulations.

It has been noted that the management of chronic diseases is made better by incorporating an education program into the management plan [23]. Also, successful diabetes self-management activities help prevent complications later, which is needed to improve their quality of life [24]. However, mere transferring of knowledge about self-care is not enough. It requires the adoption of a planned behavior change communication process, and patients should actively be encouraged to engage in self-care behavior. Our educational intervention was planned on behavior change theories to encourage subjects to participate in their diabetes management with better self-care practices. After two sessions, it was noted that there was a significant improvement in the self-care scores. While this was at the end of two months, further evaluation and regular sessions would be necessary to establish community acceptance and long-term change.

As we only studied FBS levels, including post-prandial sugar levels and HbA1C would have provided more information regarding exact glycemic control. Information about years of treatment could have provided a better perspective on self-care practices. We also feel that peer-group discussion might motivate confirmation bias. These limitations can be addressed through regular individual follow-ups and discussions of treatment goals with the patients. Regular peer-group discussions will also help build a self-care support community.

## Conclusions

This study shows that overall self-care practices were not satisfactory in most diabetic participants. However, study subjects showed compliance with regular intake of medications and foot care practices. Small group education significantly impacted the self-care practices among people with diabetes, as reflected in the pre- and post-intervention scores.

Among individuals with diabetes, participating in one's care will help to slow the disease's progression. This stresses the need to incorporate behavior change communication practices in health education sessions and awareness campaigns to control and reduce the burden of diabetes in the community. This highlights the need to focus on a community-based approach to tackle this modern epidemic as envisaged under the national programs.
